# The spread of heated tobacco product (HTP) use across various subgroups during 2015–16 and 2017–18 in Japan

**DOI:** 10.1265/ehpm.22-00219

**Published:** 2023-01-18

**Authors:** Ai Hori, Takahiro Tabuchi, Naoki Kunugita

**Affiliations:** 1 Department of Global Public Health, Faculty of Medicine, University of Tsukuba, 1-1-1 Tennodai, Tsukuba-city, Ibaraki 305-8577, Japan; 2 Cancer Control Center, Osaka International Cancer Institute, 3-chōme-1-69 Otemae, Chuo Ward, Osaka 541-8567, Japan; 3 School of Health Sciences, University of Occupational and Environmental Health, 1-1 Iseigaoka, Yahatanishi-ward, Kitakyushu-city, Fukuoka 807-8555, Japan

**Keywords:** Tobacco, Heated tobacco product, HTP, IQOS, JASTIS, Japan, Smoking, Nicotine, Tobacco survey

## Abstract

**Background:**

Heated tobacco product (HTP) use has increased substantially between 2016 and 2017 in Japan. This study aims to clarify how HTP use (IQOS, Ploom, and glo) spread across the different combustible cigarette smoking statuses during 2015–16 and 2017–18 in Japan.

**Methods:**

We compared the two periods of (i) 2015 to 2016 (N = 5,366) and (ii) 2017 to 2018 (N = 3,422) from a longitudinal study randomly sampling members from the Japan “Society and New Tobacco” Internet Survey (JASTIS). Multivariable logistic regression models for current HTP use in the previous 30 days by combustible cigarette smoking status in the previous year were used adjusting for socio-demographic factors.

**Results:**

HTP use increased by 10 times in the 2017–18 cohort compared with the 2015–16 cohort according to the adjusted odds ratio (95% confidence interval) for current HTP use as 10.2 (7.03–14.8). According to smoking status, significantly higher adjusted ORs (95% CIs) of current HTP use for the after period were observed: 2.60 (1.37–4.94) for never smokers, 7.82 (3.64–16.8) for former smokers, 21.1 (5.73–77.9) for current smokers with intention to quit, and 17.0 (9.58–30.3) for current smokers without intention to quit.

**Conclusion:**

During 2015 to 2018 in Japan, HTP use dramatically increased in all subgroups except for never smokers.

**Supplementary information:**

The online version contains supplementary material available at https://doi.org/10.1265/ehpm.22-00219.

## Background

Heated tobacco products (HTPs) are nicotine-containing products that have been sold, since their launch in 2014, without sufficient evidence as to their effect on human health. This has given rise to a rapidly growing public health concern around the world [1]. HTPs are a different type of tobacco product from cigarettes or electronic cigarettes that absorb the vapor generated by heating a dedicated stick or capsule derived from tobacco leaves with an electronic device [2]. HTP-derived vapor contains nicotine, carcinogens including formaldehyde, and glycerol but there is insufficient evidence on the effect exposure to it has on human health and safety [2, 3]. Although there has been no careful assessment of health impact, HTPs are already marketed in about forty countries as of 2019 [4]. In Japan, which is the world’s first market for HTPs and where the sale of nicotine-containing electronic cigarettes is prohibited without official approval, the use of HTPs has increased greatly since the broadcast of a TV program in 2016 [5]. After the TV promotion, HTP use among all Japanese adults increased substantially between 2016 and 2017, showing prevalence of 0.8% in 2016 and 3.7% in 2017 [6]. The increase in HTP use seems to have continued since 2016 in Japan, as shown in our inverse probability weighting estimate for the internet survey data with nationally representative data [6]. In 2021, multiple HTPs (IQOS, Ploom, and glo) have become readily available in convenience stores and drug stores and are aggressively promoted.

The facts about the current HTP user profile remain unclear, although the tobacco industry promotes the products as if HTPs were only for the use of adult smokers. In this study, we aim to describe how people with different combustible cigarette smoking statuses in 2015 started to use HTPs during the 2016 to 2017 period of substantial diffusion, by using the Japan “Society and New Tobacco” Internet Survey (JASTIS) dataset, as documented elsewhere [7]. A previous report showed that men, younger people, and cigarette smokers with intention to quit were more likely to start using HTPs in the early phase of the HTP introduction [5]. Our aim is now to confirm whether this tendency is continuing or spreading into other groups. Especially, HTP use among never smokers and former cigarette smokers needs to be carefully monitored to prevent new or resumed nicotine dependency. This study aims to clarify how HTP use spread across the different cigarette smoking statuses and intention to quit, during 2015–16 and 2017–18 in Japan.

## Methods

### Participants

We conducted a baseline internet survey between 31 January and 17 February 2015, randomly sampling members from a large panel registered with a major Japanese internet research agency, Rakuten Insight, which has 2.3 million panelists. At the time of registration, panelists were required to provide information such as sex, age, occupation, and residence and to agree that they would participate in different research surveys. The baseline survey comprised men and women aged 15–69 years (n = 8240) in 2015. Further details are available in a previous report [7]. One-year, 2-year and 3-year follow-up surveys were conducted from 29 January to 15 February 2016 and from 27 January to 27 February 2017, and from 26 January to 20 March 2018, respectively. In the follow-up surveys in 2016, 2017, and 2018, the questionnaire was mailed to individuals who had participated in the previous year’s survey; subjects who did not respond were nevertheless included in the follow-up survey.

### Exclusion criteria

To validate data quality, we excluded respondents who showed discrepancies and/or artificial/unnatural responses. Various checks were used to identify discrepancies; as question items were different each survey year, for items such as the total number of household members (2015), we used: “Please choose the second from the bottom” (2017). We also checked for participants who chose the same number throughout a set of questions (2015, 2016, 2017, and 2018). After excluding respondents with any discrepancies in 2015 (n = 815, n = 8240 remaining), we further excluded respondents with discrepancies or artificial/unnatural responses in the follow-up surveys (n = 72 for 2016; n = 90 for 2017; and n = 257 for 2018).

**Fig. 1:**
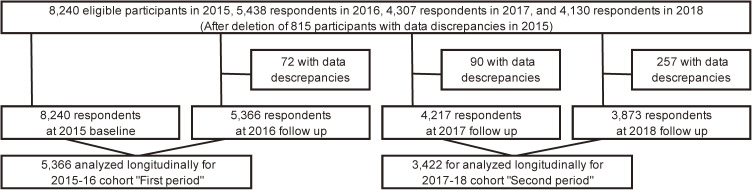
Flow chart of participants selection for the analyses

We then merged the two-year datasets; 2015 to 2016, and 2017 to 2018, and analyzed the resulting 5,366 individuals as “First period (2015–16)” and 3,422 individuals as “Second period (2017–18)” (Fig. 1). We compared the two periods, 2015–16 and 2017–18 because our analysis aims to evaluate how HTP use spread among each subgroup before and after a substantial HTP use increase between 2016 and 2017. The second period (2017–18 cohort) participants were respondents from the same cohort, except for a few participants (n = 183) who did not participate in the 2016 survey or who had data discrepancies in 2016.

### Cigarettes smoking status and intention to quit smoking

We defined current smoker as combustible cigarette (tailor-made and/or hand-rolled cigarettes) user in the previous 30 days. “Former smokers” were participants who had habitually used combustible cigarettes before but do not use them currently. For the 2015–16 period, participants were asked: “Are you currently smoking or using cigarettes?” for tailor-made and hand-rolled cigarettes. We defined “Never smoker” as participants who answered, “I have never smoked cigarettes” or “I tried them more than once but did not use them habitually”, “Former smoker” as “I used them habitually before but I have now stopped”, and “current smoker” as “I occasionally use them” or “I use them almost every day”. For the 2017–18 period, we asked “Have you used cigarettes within the last 30 days?” for tailor-made and hand-rolled cigarettes. We defined “never smoker” as participants who answered, “I did not use them within the last 30 days” and answered as “never smokers” in 2015. “Former smokers” were defined as participants who answered, “I did not use them within the last 30 days” and who had answered as current or former combustible cigarette smokers in 2015. We defined respondents who answered, “I use them occasionally” and “I use them almost every day” as “current combustible cigarette smoker” in 2017.

“Current smokers with an intention to quit” were current smokers who reported intending to quit smoking within 6 months or within 1 month. “Current smokers without intention to quit” included participants who did not intend to quit or intended to quit but not within 6 months.

### HTP use in previous 30 days

Panelists were asked about their current use (previous 30 days) of each product (Ploom, IQOS, and glo) in 2015, 2016, 2017 and 2018 surveys (glo was not included until 2017 and 2018, because it did not enter the market until December 2016). The term ‘Ploom TECH’ was used instead of Ploom in 2017 and 2018, following a product name change. Respondents who answered “yes” to the question: “Have you used the following products in the previous 30 days?” (options: Ploom, IQOS, and glo) were defined as current users of the designated products [5, 8, 9].

### Covariates

To determine education level, participants were asked: “Please choose the level of school from which you graduated (or dropped out) or where you are currently enrolled; junior high school, high school, junior college, technical college, university, or graduate school”. For the number of family members, we used the question: “How many household members do you have, who are living together (including you)?”, then categorized responses as: “single” or “two or more”. To determine self-rated health, we asked, “Please choose your current state of health: 1: very good, 2: good, 3: ordinary, 4: poor, 5: very poor” and categorized very good, good, and ordinary, as “good” and poor and very poor as “poor”. We used the area-level deprivation index (ADI) to capture the geographical accumulation of deprived populations living in a given postal district (cho-aza level) [10]. Briefly, ADI is a continuous variable calculated by eight census variables related to deprivation (i.e., the proportion of old couple households, old single households, single-mother households, rental houses, sales and service workers, agricultural workers, blue-collar workers, and unemployment rate) [11]. We divided the aggregated postal districts into quartiles based on ADI; higher quartiles represent more disadvantaged neighborhoods.

### Statistical analyses

We conducted a logistic regression analysis which predicts 2016 HTP use in the previous 30 days by smoking category in the previous year (e.g., 2015 for 2015–16 cohort) (see Additional file 1 Table S1). The multivariable model was adjusted for gender (men or women), age group (5 categories; 15–29, 30–39, 40–49, 50–59, 60 years or more), educational level (less than high school, or technical school/college/university or more), family members (single or more than one), self-rated health (poor or good), and areal deprivation index (ADI; 1st quartile – least deprived to 4th quartile – most deprived). Analyses were repeated for the 2017–18 survey period.

Pooling 2015–16 cohort and 2017–18 cohort data, multivariable logistic regressions with generalized estimating equation (GEE) were conducted to estimate the odds ratios for the 2017–18 cohort (second period) compared with the 2015–16 cohort (first period). Adjusted odds ratios were estimated for the comparison of HTP use prevalence in 2018 (2017–18 cohort) with 2016 (2015–16 period). GEE was conducted in each subgroup (stratified analysis) to examine the differential impact of the diffusion of HTP use between the first and second periods with multivariate adjusting. P values for the interaction term between the period and each variable were calculated to test whether the period had the same effect for each category. Statistical tests were two-sided and regarded as statistically significant at P value < 0.05. All analyses were done using Stata MP version 15.0 (Lakeway Drive College Station, TX, USA).

## Results

Table 1 shows the prevalence of current HTP use according to characteristics in 2015–16 cohort (first period) and 2017–18 cohort (second period). Overall prevalence of current HTP use was 0.7% in 2016, and 5.6% in 2018. Prevalence of current HTP use among cigarette smokers with intention to quit in the previous year was 2.6% in first period and increased to 32.8% afterwards. Among cigarette smokers without intention to quit in the previous year, prevalence of current HTP use in first and second period was 2.0% and 24.2%, respectively. Among former combustible cigarette smokers in the previous year, prevalence of current HTP use was 0.7% in first period and increased to 4.4% afterwards. The first and second ratio, i.e., HTP use in 2017–18 (%) divided by the status in 2015–16 (%), was more than five, among all the subgroups, except for never smokers, although these estimates were not adjusted for other variables.

**Table 1:** Prevalence of current use of heated tobacco products by characteristics, first and second survey period

Characteristics*	**First period** **(2015–16 cohort)**		**Second period** **(2017–18 cohort)**		**First and ** **Second ** Ratio**

	**No. of ** **participants**	**Current use ** **(%) (1)**	**No. of ** **participants**	**Current use ** **(%) (2)**	
**Total**	5366	37 (0.7)	3422	192 (5.6)	8.0
**Gender**					
Men	2762	28 (1.0)	1847	147 (7.9)	7.9
Women	2604	9 (0.4)	1575	45 (2.9)	7.3
**Age (years)**					
15–29	1036	14 (1.4)	326	22 (6.8)	4.9
30–39	961	10 (1.0)	616	45 (7.3)	7.3
40–49	1093	6 (0.6)	762	50 (6.6)	11.0
50–59	1092	5 (0.5)	781	59 (7.6)	15.2
60 or more	1184	2 (0.2)	937	16 (1.7)	8.5
**Smoking status**					
Never smoker	3767	15 (0.4)	2247	19 (0.9)	2.3
Former smoker	833	6 (0.7)	615	27 (4.4)	6.3
Smoker with intention to quit	115	3 (2.6)	122	40 (32.8)	12.6
Smoker without intention to quit	651	13 (2.0)	438	106 (24.2)	12.1
**Education**					
Less than high school	2172	16 (0.7)	1295	80 (6.2)	8.9
Technical school, college, university or more	3175	21 (0.7)	2123	112 (5.3)	7.6
**Family members**					
Single	880	10 (1.1)	556	34 (6.1)	5.5
Two or more	4486	27 (0.6)	2866	158 (5.5)	9.2
**Self-rated health**					
Poor	638	4 (0.6)	373	17 (4.6)	7.7
Good	4728	33 (0.7)	3049	175 (5.7)	8.1
**Area-level deprivation index of residence**					
1st quartile (least deprived)	1323	12 (0.9)	859	41 (4.8)	5.3
2nd quartile	1371	9 (0.7)	896	55 (6.1)	8.7
3rd quartile	1289	9 (0.7)	808	48 (5.9)	8.4
4th quartile (most deprived)	1296	6 (0.5)	813	44 (5.4)	10.8

HTP, heated tobacco product;

*Characteristics in 2015 and 2017 indicated for first and second period, respectively;

**Second/First = (2)/(1) unadjusted ratio of HTP use.

Note: Current use means HTP use prevalence in 2016 for the first period and 2018 for the second period. Missing values exist for education (19 in 2015–16 and 4 in 2017–18) and area-level deprivation index of residence (87 in 2015–16 and 46 in 2017–18).

HTP and cigarette dual use increased over the period among current combustible cigarette smokers, but not among former smokers and never smokers (Table 2).

**Table 2:** Smoking status transition by initial smoking category in first (2015–16) and second (2017–18) survey period

Initial smoking category* **Smoking status in the following year**	**First period** **(2015–16 cohort)**		**Second period** **(2017–18 cohort)**	

	**No. of participants**	**(%)**	**No. of participants**	**(%)**
**Never smoker**	3767		2247	
Current non smoker	3618	(96.0)	2201	(98.0)
Cigarette user	134	(3.6)	27	(1.2)
HTP user	6	(0.2)	9	(0.4)
Dual user	9	(0.2)	10	(0.5)
**Former smoker**	833		615	
Current non smoker	778	(93.4)	548	(89.1)
Cigarette user	49	(5.9)	40	(6.5)
HTP user	4	(0.5)	16	(2.6)
Dual user	2	(0.2)	11	(1.8)
**Smoker with intention to quit**	115		122	
Current non smoker	22	(19.1)	32	(26.2)
Cigarette user	90	(78.2)	50	(41.0)
HTP user	0	(0.0)	8	(6.6)
Dual user	3	(2.6)	32	(26.2)
**Smoker without intention to quit**	651		438	
Current non smoker	62	(9.5)	23	(5.3)
Cigarette user	576	(88.5)	309	(70.6)
HTP user	1	(0.2)	34	(7.8)
Dual user	12	(1.8)	72	(16.4)

*Initial smoking category means the smoking status in 2015 for the first period and 2017 for the second period.

Table 3 shows the odds ratios (OR) (95% confidence intervals [95% CI]) for current HTP use according to the previous year’s characteristics. Adjusted OR (95% CI) for the first and the second period were 3.02 (1.09–8.40) and 7.29 (3.87–13.8) for former combustible cigarette smokers, 7.57 (2.07–27.6) and 58.9 (31.4–110) for current smokers with intention to quit, and 6.19 (2.75–13.9) to 43.5 (25.3–74.9) for current smokers without intention to quit, respectively, compared with never smokers (reference category). Compared with 15–29 years (reference category), older people were less likely to use HTPs: the OR (95% CI) first and second period were 0.61 (0.26–1.42) and 0.90 (0.47–1.75) for 30–39 years, 0.26 (0.10–0.72) and 0.46 (0.24–0.89) for 40–49 years, 0.22 (0.07–0.64) and 0.53 (0.28–0.99) for 50–59 years, and 0.09 (0.02–0.40) and 0.13 (0.06–0.27) for 60 years or more.

**Table 3:** Logistic regression models for current use of heated tobacco products (HTPs)

Characteristics*	**Odds Ratio (95% Confidence Interval) of HTP use**			

	**First period (2015–16 cohort)**		**Second period (2017–18 cohort)**	

	**Unadjusted**	Adjusted**	**Unadjusted**	**Adjusted**
**Cigarette smoking status**				
Never smoker	Reference	Reference	Reference	Reference
Former smoker	1.81 (0.70–4.69)	3.02 (1.09–8.40)^†^	5.38 (2.97–9.75)^†^	7.29 (3.87–13.8)^†^
Smoker with intention to quit	6.70 (1.91–23.5)^†^	7.57 (2.07–27.6)^†^	57.2 (31.7–103)^†^	58.9 (31.4–110)^†^
Smoker without intention to quit	5.10 (2.41–10.8)^†^	6.19 (2.75–13.9)^†^	37.4 (22.7–61.8)^†^	43.5 (25.3–74.9)^†^
**Gender**				
Men	Reference	Reference	Reference	Reference
Women	0.34 (0.16–0.72)^†^	0.49 (0.23–1.09)	0.37 (0.26–0.52)^†^	0.68 (0.46–1.003)
**Age**				
15–29	Reference	Reference	Reference	Reference
30–39	0.77 (0.34–1.74)	0.61 (0.26–1.42)	0.99 (0.58–1.67)	0.90 (0.47–1.75)
40–49	0.40 (0.15–1.05)	0.26 (0.10–0.72)^†^	0.91 (0.54–1.51)	0.46 (0.24–0.89)^†^
50–59	0.34 (0.12–0.94)^†^	0.22 (0.07–0.64)^†^	1.10 (0.67–1.81)	0.53 (0.28–0.99)^†^
60 or more	0.12 (0.03–0.54)^†^	0.09 (0.02–0.40)^†^	0.26 (0.14–0.48)^†^	0.13 (0.06–0.27)^†^
**Education level**				
Less than high school	Reference	Reference	Reference	Reference
Technical school, college, university or more	0.90 (0.47–1.72)	0.91 (0.45–1.81)	0.80 (0.59–1.07)	1.06 (0.75–1.49)
**Family members**				
Single	Reference	Reference	Reference	Reference
Two or more	0.53 (0.25–1.09)	0.78 (0.36–1.69)	1.01 (0.68–1.51)	1.16 (0.74–1.81)
**Self-rated health**				
Poor	Reference	Reference	Reference	Reference
Good	1.11 (0.39–3.16)	1.09 (0.38–3.13)	1.13 (0.69–1.83)	1.15 (0.65–2.06)
**Area-level deprivation index of residence**				
1st quartile (least deprived)	Reference	Reference	Reference	Reference
2nd quartile	0.72 (0.30–1.72)	0.70 (0.29–1.68)	1.29 (0.86–1.94)	1.15 (0.72–1.84)
3rd quartile	0.77 (0.32–1.83)	0.78 (0.32–1.88)	1.17 (0.77–1.79)	1.60 (0.99–2.61)
4th quartile (most deprived)	0.51 (0.19–1.36)	0.49 (0.18–1.32)	1.06 (0.69–1.64)	1.25 (0.76–2.05)

Note: HTP use in 2016 for the first period and 2018 for the second period. HTP, heated tobacco product.

*Characteristics in 2015 and 2017 indicated for each period, respectively.

**Adjusted for all listed variable.

^†^Statistically significant (p < 0.05).

**Fig. 2:**
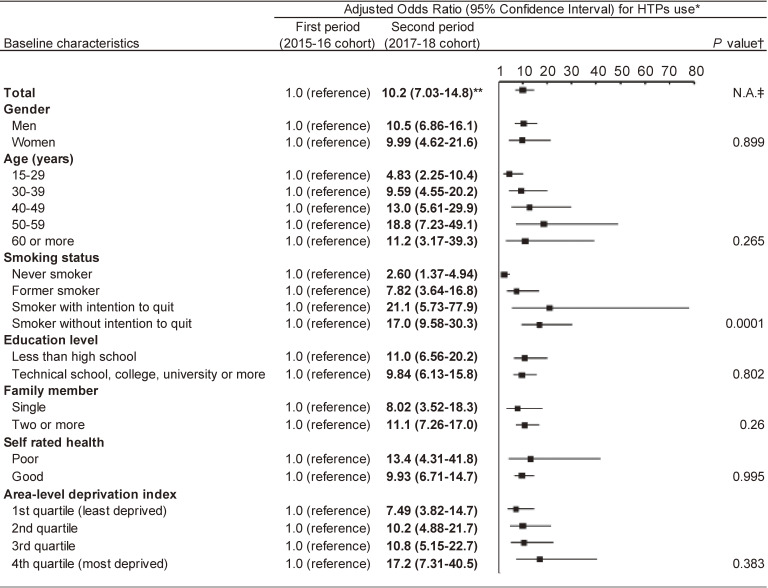
Stratified analysis of logistic regression with a generalized estimating equation modification for heated tobacco products Note: The figure shows result for 2017–18 cohort (second period) compared to 2015–16 cohort (first period). HTPs use means HTP use prevalence in 2016 for the first period and 2018 for the second period.; *Logistic regression with a generalized estimating equation modification, adjusting for all listed variables in addition to the cohort variable (before or after); **Boldface indicates statistically significance (p < 0.05); ^†^P values for the interaction term between the period and each variable. ^‡^N.A., not applicable

Results of the odds ratios of current HTP use for the 2017–18 cohort (second period) compared with the 2015–16 cohort (first period) are shown in Fig. 2. Among the total sample, adjusted OR (95% CI) for current HTP use for the after period was 10.2 (7.03–14.8). Analyzing by smoking status, significantly higher adjusted ORs (95% CIs) of current HTP use for the after period were observed: 2.60 (1.37–4.94) for never smokers, 7.82 (3.64–16.8) for former combustible cigarette smokers, 21.1 (5.73–77.9) for current smokers with intention to quit, and 17.0 (9.58–30.3) for current smokers without intention to quit. Adjusted ORs of current HTP use in the second period were more than five and statistically significant among all characteristic subgroups, except for never smokers (P = 0.0001 interaction term was significant for smoking status only).

## Discussion

This report shows the rapid spread of HTP use after the initial diffusion in 2016 in Japan. The prevalence of HTP use increased in a short period, from 0.2% in 2015 to 11.3% in 2019 [6]. This increase was confirmed by multivariable logistic regression with adjusted estimates: HTP use increased by about 10 times in the second period compared with the first period according to the adjusted odds ratio (95% confidence interval) for HTP use of 10.2 (7.03–14.8). The present study also shows that HTP use spread over all categories of gender, age, cigarette smoking status, educational level, household composition, self-rated health, and area-level deprivation index, except for never smokers. In Japan, HTPs were approved for sale in 2014 and their use has spread widely since 2016 without sufficient scientific evidence about the associated health risks. This was before the World Health Organization and a number of governments suggested that HTPs should be regulated as a tobacco product [4, 12].

Surprisingly, HTP use has already spread to 1 in 3 current cigarette smokers with intention to quit, and 1 in 4 current smokers without intention to quit. Due to the widespread availability of HTPs, cigarette smokers who find it difficult to quit smoking completely may replace cigarettes with other tobacco products including HTPs. HTPs might therefore deprive smokers of the opportunity to stop using tobacco altogether which would reduce tobacco-attributable deaths [13] such as cardiovascular diseases, respiratory diseases, and neoplasms [14]. HTP penetration in Japan is an obstacle to complete smoking cessation.

In addition, this is also the first report to show the considerable increase in HTP use among former combustible cigarette smokers, as the adjusted odds ratio (95% confidence interval) increased from 3.02 (1.09–8.40) in 2015–16 to 7.29 (3.87–13.8) in 2017–18. Previous studies reported the prevalence of HTP ever use as 0.82% in the UK [15], 1.4% in Italy [16], 2.2% in the US [17] and 4.4% in South Korea [18]. All these studies found a positive association between HTP use and current cigarette smoking, being male, and being younger. Because young, male smokers might “perceive relative advantage [19]” of HTPs, they were understood to be “early adopters” who contributed to the adoption rate of HTPs. Previous studies on electronic cigarettes (e-cigarettes) in North America and European countries showed that being male, younger, and a current or former smoker were significant predictors for e-cigarette use in the diffusion phase [20, 21]. In line with these previous findings, the characteristics of HTP users in the present study were similar to those in South Korea and those of e-cigarette users in Western countries. The increase in HTP use among respondents who were former combustible cigarette smokers in the previous year may suggest that HTPs have the potential to encourage resumption of nicotine dependency for former smokers.

Our experience in Japan – which has the highest HTP use in the world– may be helpful for predicting the spread of HTP use in other countries. The HTP diffusion resulted from aggressive marketing strategies mounted by the tobacco industry [22]. In Japan, HTPs are widely sold in convenience stores, drug stores, vending machines, and websites accompanied by aggressive advertising. For example, the IQOS advertisement announces, “IQOS vapor reduces the amount of harmful components by about 90%” [23], in a large font, while in a very small font it adds “This does not mean that the adverse effect on the health of this product is small compared to other products.” Advertising regulation for HTPs in Japan is based on the Ministry of Finance notification [24] and self-regulation by the tobacco industry, the same as combustible cigarettes. The widespread distribution of HTPs might make it difficult to create public health policies for a tobacco-free society. Following Framework Convention on Tobacco Control (FCTC) recommendations [25], policymakers need to ban HTP marketing by the tobacco industry and provide appropriate warning messages about HTPs as well as conventional cigarettes as HTP use has already reached considerable proportions in Japan.

### Limitations

This study has several limitations. First, some subjects were lost to attrition. For example, young people, who use HTPs more than the older participants, tended to drop out. Therefore, the prevalence of HTP use may have been underestimated in the follow-up study, although we found significant initiation of HTP use not only among the younger population but in every age category. Second, characteristics including smoking status or household membership could change at any time throughout the study period. Participants who were categorized as former smokers in the study may have used HTPs when they were current smokers: i.e., we do not know whether former smokers used HTPs before or after they stopped cigarette smoking [17]. In the present study, 3.4% (19/559) of former smokers in the 2015 baseline become HTP users in 2018, indicating a relatively consistent result with Table 1 (4.4%). In addition, this study did not distinguish between trial HTP use and habitual use because we focused on the first step of HTP use among the Japanese population. Longitudinal analyses to assess the effect of HTP use on cigarette smoking cessation will be necessary in the future. Further long-term monitoring should be done to examine how HTP use spreads across different smoking statuses.

## Conclusions

HTP use in Japan spread rapidly and widely to all groups following the initial diffusion in 2016. HTP use has increased not only among current cigarette smokers but also among former smokers. There is therefore a risk that HTP use might encourage the resumption of tobacco use.

## Abbreviations

HTP: Heated Tobacco Products
JASTIS: Japan “Society and New Tobacco” Internet Survey
CI: Confidence Interval
ADI: Area-level Deprivation Index
GEE: Generalized Estimating Equation
OR: Odds ratios
FCTC: Framework Convention on Tobacco Control

## Supplementary information

**Additional file 1:** **Table S1.** Survey year of variables by cohorts, first and second survey period.

## Declarations

### Availability of data and materials

Deidentified study data may be made available on request to researchers who submit a proposal that is approved by the principal investigator (TT).
