# Supplementary material for: The spread of heated tobacco product (HTP) use across various subgroups during 2015–16 and 2017–18 in Japan
Source: Environ Health Prev Med. 2023 Jan 18;28:5. doi: 10.1265/ehpm.22-00219 (PMC9884561; doi:10.1265/ehpm.22-00219)
Supplement: Supplementary file 1 — Additional file 1: Table S1. Survey year of variables by cohorts, first and second survey period. [file ehpm-28-005-s001.pdf]

Table S1 shows the survey year of variables separately for cohorts in the first and second survey period.

Table S1. Survey year of variables by cohorts, first and second survey period

Variables*	First period (2015-16 cohort)		Second period (2017-18 cohort)	
	2015	2016	2017	2018
Gender	✓		✓	
Age	✓		✓	
Smoking status	✓		✓	
Education	✓		✓	
Family members	✓		✓	
Self-rated health	✓		✓	
Area-level deprivation index of residence	✓		✓	
HTP use		✓		✓

HTP, heated tobacco product
